# Effect of a Blend of Essential Oils, Bioflavonoids and Tannins on In Vitro Methane Production and In Vivo Production Efficiency in Dairy Cows

**DOI:** 10.3390/ani12060728

**Published:** 2022-03-14

**Authors:** Carlo Angelo Sgoifo Rossi, Silvia Grossi, Matteo Dell’Anno, Riccardo Compiani, Luciana Rossi

**Affiliations:** Department of Health, Animal Science and Food Safety “Carlo Cantoni” (VESPA), Università degli Studi di Milano, 26900 Lodi, Italy; carlo.sgoifo@unimi.it (C.A.S.R.); matteo.dellanno@unimi.it (M.D.); riccardo.compiani@gmail.com (R.C.); luciana.rossi@unimi.it (L.R.)

**Keywords:** sustainability, methanogenesis, natural alternatives, efficiency, dairy cows

## Abstract

**Simple Summary:**

The dairy system is facing many environmentally related issues, such as green-house gas emissions (GHG), as well as an increased demand for milk by the growing world population. Dairy cow farming must evolve towards more efficient and sustainable ways of production. Strategies to reduce ruminal methane emissions must be considered, due both to methane’s direct involvement in global warming and its negative relationship with productivity. Besides being the most important GHG arising from dairy cows, methane is also correlated with a loss of energy and a reduction in production efficiency that further worsen the environmental impact. The use of natural extracts, such as essential oils, bioflavonoids, and tannins may be useful to reduce methane production and to modulate the ruminal microbiota toward more efficient fermentation, increased feed efficiency and improved overall productivity.

**Abstract:**

Two trials were performed to evaluate the efficacy of a blend of essential oils, bioflavonoids and tannins on methane (CH_4_) emissions (in vitro) and on the production efficiency of dairy cows (in vivo). The in vitro trial tested the production of total gas and CH_4_ at 16, 20 and 24 h of incubation, and volatile fatty acids (VFA) at 16 and 24 h, through biochemical methane potential (BMP) assays. In the in vivo trial, milk yield, dry matter intake (DMI), feed conversion rate (FCR), milk quality and apparent total tract digestibility (aTTD) were evaluated in 140 lactating Holstein Friesian cows. Animals were allocated into two groups: (i) Control, standard diet; (ii) Treatment, standard diet plus 10 g/head/d of a powder with a 10% concentration of a blend of essential oils, bioflavonoids and tannins. Statistical analysis was performed using the mixed procedure of SAS either for single or repeated measures. For all the parameters a *p*-value ≤ 0.05 was considered statistically significant. The blend significantly reduced the in vitro total gas and CH_4_ emissions at 16, 20 and 24 h of incubation (*p* < 0.001). In addition, acetic acid was reduced (*p* < 0.001), while propionic acid concentration was increased (*p* < 0.001) at 16 h and 24 h. In the in vivo trial, the Treatment group showed significantly raised milk yield, DMI, FCR (*p* < 0.001), and of the aTTD of cellulose and starch (*p* ≤ 0.002), while the milk quality traits were not affected. Overall, the results from the study indicated that the blend of essential oils, bioflavonoids, and tannins significantly reduced in vitro total gas and CH_4_ production and improved the production efficiency of lactating dairy cows in vivo.

## 1. Introduction

The livestock system is constantly criticized in various respects related to environmental sustainability, such as deforestation, water and land use, pollution, and consumption of human-edible resources, while the demand for animal-derived foods is continuously increasing. Greenhouse gas (GHGs) emissions are one of the main focal points, with livestock emissions accounting for 14.5% of the total [1,2,3].

Among food-producing animals, cattle are often the most discussed for their high contribution to the sector’s GHG emissions (35% and 32% of the total for beef and dairy, respectively), especially of methane (CH_4_) [4,5].

In response to these concerns, more efficient and sustainable dairy production systems need to be developed. Strategies to directly reduce ruminal CH_4_ production must be considered. Indeed, almost 71% of the total CH_4_ produced originates inside the rumen [3]. Specifically, digestion and fermentation of forages are related to a higher production of metabolic hydrogen (H_2_) in the rumen, which is subsequently converted to CH_4_ as a protective mechanism [6]. In addition to being a cause of environmental impacts, CH_4_ production is also an energy loss, that reduces nutrient utilization efficiency by 2 to 12%, and, in consequence, potential productivity [6,7], resulting in a negative environmental impact due to a lower yield of final products in relation to the GHGs produced [2].

Current strategies include changes in diet formulation, increasing the amount of lipids and including compounds with antimicrobial abilities, such as bacteriocins or ionophores [6,7,8,9]. Although these strategies can have a direct impact on methanogenesis, they each come with limitations with respect to animal efficiency, diet digestibility and the need to reduce antimicrobial use [6]. Antimicrobials, such as monensin sodium, an ionophore effective in the reduction of methanogenesis, have been banned for non-therapeutic purposes in the European Union from 2006 [10,11]. In this context, safer compounds with anti-methanogenic properties are needed.

One of the novel alternatives that has shown promise is the use of natural additives, such as essential oils, bioflavonoids, and tannins [8]. Bioflavonoids and tannins are natural polyphenolic compounds widely present in plants which are used in animal nutrition due to their antioxidant, anti-inflammatory and antimicrobial properties [12,13,14]. Essential oils are plant metabolites consisting of phenylpropenes and terpenes with proved antioxidant properties and strong antimicrobial capacity toward different categories of microorganisms and bacteria and are currently being studied in relation to animal nutrition [15].

In ruminants, essential oils and other natural compounds are being investigated as potential modifiers of ruminal fermentation to improve nutrient utilization efficiency and to reduce methane production [7,8,16]. Although the effects and mode of action are variable depending on their molecular characteristics, they have shown an inhibitory effect on Gram-positive bacteria and consequently on H_2_ production and methanogenesis [17,18]. Different studies, conducted either in vitro or in vivo, have demonstrated the efficacy of essential oils in reducing CH_4_ production while increasing the production of volatile fatty acids and digestion efficiency [19,20,21,22,23,24,25].

Coriander oil has been reported to potentially modulate in vitro digestibility and CH_4_ production [26]. Eugenol (4-allyl-2-methoxyphenol), a phenolic monoterpene present in high quantities in clove (*Syzygium aromaticum*) bud, and cinnamon (*Cinnamomum cassia*), has demonstrated antimicrobial activity against Gram-positive and Gram-negative bacteria [27,28]. In addition, Geranium essential oils, produced by steam distillation of leaves of various *Pelargonium* species, have been reported to have antibacterial effects [29]. Previous experiments performed with a combination of coriander and eugenol essential oils, and geranyl acetate, both in vitro and in vivo on dairy cows, have highlighted positive results on CH_4_ production, milk yield and quality, and feed conversion rate, depending on the dosage and duration of administration [8,18,19,21,30]. However, in vivo results in dairy cows are highly variable, probably due to the different classes of essential oils, dosage, and duration of administration [8,16,25].

The aim of the present study was to evaluate the efficacy of a coated blend of coriander seed oil, eugenol and geranyl acetate essential oils, combined with bioflavonoids and tannins, in reducing CH_4_ production in vitro and in improving the production performance and apparent total tract digestibility of the diet in vivo using lactating Holstein dairy cows.

## 2. Materials and Methods

Two different trials were set up to evaluate the efficacy of a coated blend of essential oils (EO), mainly from cloves (*Syzygium aromaticum*), coriander seed (*Coriandrum sativum*), and geranium (*Pelargonium cucullatum*), tannins (CT) from chestnuts (*Castanea sativa*) and bioflavonoids (BF) from olives (*Olea europea*) (Anavrin, Vetos Europe SAGL, via delle Industrie 18, 6593—Cadenazzo, Switzerland). The relative concentrations of the active principles in the product were: EO:CT:BF = 1:2.5:0.1. The blend was tested in vitro to evaluate its potential activity in reducing CH_4_ production under standardized conditions. Then, the blend was tested in vivo using lactating dairy cows to evaluate its effects on milk production and the apparent total tract digestibility of the diet.

### 2.1. Trial I: Study of the Effect of the Blend of Essential Oils, Bioflavonoids and Tannins on In Vitro CH_4_ Production

In vitro CH_4_ production was evaluated using the biochemical methane potential (BMP) assay methodology, following the official guidelines, as reported in ISO 15985 (2004), ISO 14853 (1998), UNI/TS 11703 (2018) Hollinger et al. [31] and Agelidaki et al. [32]. The total gas (CH_4_; carbon dioxide—CO_2_; hydrogen sulphide—H_2_S; trace nitrogen; carbon monoxide—CO) concentrations were evaluated after 16, 20 and 24 h, using a water displacement method, as reported by Sarker et al. [33]. Quantification of CH_4_ was conducted through gas chromatography.

Two separate assays were set up to test the effect of a blend of essential oils, bioflavonoids and tannins, in different forms (powder and liquid), in different concentrations, at different time points and in comparison to monensin sodium.

#### 2.1.1. Common Methodology

A total of 10 close reactors, maintained in anaerobic conditions at constant temperature (37 °C) for the entire testing period of each assay, were used in each assay.

Anaerobic mud, taken from an anaerobic digester of cattle slurry, was used as an inoculum to provide the microbial population as well as the main nutrients [31]. The anaerobic mud was preventatively acclimatized at 35 °C for 48 h.

Anhydrous glucose was used as a substrate due to its standardized and well-known potential for CH_4_ production [31,32]. The total quantities added (glucose plus the blend) were defined with reference to the reactor final volume (759 mL), glucose concentration in the reactor (2.7 g/L), and the ratio between glucose and the inoculum (0.25), that needed to be considered for preparation of the reactor. The required dosage of glucose, equal to 2000 mg for all the tests, was administered by adding in each reactor 20 mL of a solution at 10% of anhydrous glucose.

The blend was added to the glucose in different percentages, as reported in Table 1 and Table 2. The quantities were lower in the second assay due to a higher concentration of the blend in the liquid form. Considering the small quantities of the blend, diluted solutions were prepared.

At each timing (16, 20 and 24 h), the total gas production was evaluated using acidified water to reduce CO_2_ solubility [33]. The total gas production was expressed as normal cubic meters (Nm^3^) per ton of substrate. The gas was then evaluated using gas chromatography to determine CH_4_ production, expressed in normal cubic meters (Nm^3^) of CH_4_ per ton of substrate, and as the percentage of total gas [34].

#### 2.1.2. Specificity of the Two Assays

In the first assay, the blend was in powder form, with a concentration of 10%. The assay consisted of five different tests, performed in replicates (as reported in Table 1), one without any addition of the blend (Control Test 1), and the others (Tests 2 to 5) with four different percentages of inclusion on a substrate dry matter (DM) basis. The total gas and CH_4_ production were evaluated after 20 h of incubation.

In the second assay, the blend was pure and in liquid form. The assay consisted of five different tests, performed in replicates, as reported in Table 2, one without any addition (Control Test 1), a second with the addition of monensin sodium with a concentration of 20% of the active ingredient (Test 2), and the others (Tests 3 to 5) with different concentrations of the blend on a substrate DM basis. The total gas and CH_4_ production were evaluated after 16 h and 24 h of incubation. Samples of the digestate were collected to analyse the concentrations of volatile fatty acids (VFA) using mass gas chromatography (MP 58C/2018 rev. 0).

### 2.2. Trial II: In Vivo Study of the Effect of a Blend of Essential Oils, Bioflavonoids and Tannins on Dairy Cow Performance

#### 2.2.1. Animals, Groups and Animal Care

The study was performed in an intensive dairy farm, the Del Santo farm (Castelgerundo, Lodi, Italy). A total of 140 lactating Holstein Friesian cows were randomly allotted to two groups blocked per days of lactation and number of lactations and followed for 100 days: (i) Control (*n* = 70; days of lactation 53.85 ± 25.36; lactation 2.30 ± 0.68); (ii) Treatment (*n* = 70; days of lactation 51.33 ± 24.37; lactation 2.30 ± 0.67). The animals were reared under the same environmental conditions, in the same free housing barn on a concrete floor with straw-bedded cubicles. Cows were milked twice a day (morning 07:00 a.m.; evening 05:00 p.m.) in a herringbone milking parlor (8 + 8).

#### 2.2.2. Diets and Feeding Management

The two groups received the same diet (Table 3), formulated to satisfy the requirements for all nutrients, as reported by the NASEM (National Academies of Sciences, Engineering, and Medicine) [35]. The diet was administered ad libitum in the form of total mixed ration (TMR). The two groups differed with respect to inclusion in the mineral mix, in the Treatment group, of 10 g/head/d of a powder containing 10% of the blend of essential oils, bioflavonoids and tannins, and 90% of wheat bran, to guarantee 0.04 g/head/day of the pure blend per kg DM of the diet. In the mineral mix of the Control group, 10 g/head/d of wheat bran were included as placebo. A mixer wagon (Sgariboldi Grizly 71.26/2, capacity 26 m^3^ mixing system with two vertical augers), equipped with a balance designed to weigh both the individual ingredients and the unloaded TMR, was used to distribute the TMR once per day during the morning. Animals had free access to water for the entire experimental period.

#### 2.2.3. Parameters Recorded

##### Production Performance: Milk Yield, Feed Intake, Feed Conversion Rate, Milk Quality

The milk yield (kg/head/d) was recorded daily during milking procedures. The group average daily dry matter intake (DMI) was assessed by weighing the administered feed and refusal in the manger after 24 h, correcting for the dry matter of the diet. The feed conversion ratio (FCR) was calculated based on DMI and milk yield data. The milk quality was evaluated monthly for protein, fat, lactose, urea and somatic cell counts by the Lombardy Regional Breeders Association (ARAL) laboratory, using the Milkoscan TM FT 6500 Plus instrument (Foss, Hillerød, Denmark), employing the Fourier transform infrared spectroscopy (FTIR) measuring principle. Milk urea levels were determined using a colorimetric kit following the manufacturer’s instructions (Urea Assay Kit Rapid—K-URAMR—Megazyme—Astori Tecnica s.n.c. Poncarale (BS) 25020). The energy-corrected milk (ECM) was calculated comparing the values of protein, fat and average milk production of the same week. The ECM was obtained according to the Tyrrel and Reid (1965) equation as follows [36]:

ECM=0.327×Milk yield (kg)+12.95×Fat yield (kg)+7.2×Protein yield (kg)

The health status of animals was monitored daily by the farm veterinary staff.

##### Characteristics of the Diet, Feces and Apparent Total Tract Digestibility

Experimental diets and feces were analyzed twice per month, at the beginning and end of each month, by portable near-infrared spectroscopy (NIR) equipment (Polispec, IT Photonics, Italy).

The characteristics of the TMR were evaluated on the fresh feed, measuring three different sections along the length of the feed bunk (beginning, middle and end). Feces composition was assessed on a pool of fecal material per group collected the day after each TMR analysis. Feces of 20 cows per group were sampled from the rectal ampulla. Fecal samples from each group were pooled and mixed prior to analysis. NIR was used to measure the content of dry matter (DM), crude protein (CP), crude fats (CF), neutral detergent fiber (NDF), acid detergent fiber (ADF), acid detergent lignin (ADL), ash and starch. The cellulose content was obtained by subtracting ADF from ADL, and hemicelluloses was calculated by subtracting NDF from ADF. Sugars and pectin were obtained according to the following formula: 100 − (ash + CF + CP + NDF + starch). The apparent total tract digestibility (aTTD) was then calculated as follows, excluding the digestibility of CP, since urine data were not provided by nitrogen balance trial:

$$\mathrm{aTTD}\;\%=\frac{\left(\frac{\mathrm{Xd}}{\mathrm{ADLd}}\right)-\left(\frac{\mathrm{Xf}}{\mathrm{ADLf}}\right)}{\left(\frac{\mathrm{Xd}}{\mathrm{ADLd}}\right)}\times 100$$

where:

X = each analytical parameter considered (%)
ADL = acid detergent lignin (%)
d = diet
f = feces.

### 2.3. Statistical Analysis

Statistical analysis was performed using SAS software (SAS 9.4, SAS Institute, Cary, NC, USA). Normal distribution (data distribution and homogeneity of variances) of the obtained data was evaluated using PROC UNIVARIATE. Considering the in vitro trial, data from the first assay were evaluated using the mixed procedure of SAS. The model used included the different tests as the main effect and the reactor as a random effect. Data from the second assay were evaluated using a mixed model for repeated measures that accounted for the fixed effects of the different tests, the time of measurement, their interaction and the random effects of the reactor within treatment period.

Considering the in vivo trial, zootechnical performance (milk yield, feed intake, feed conversion rate, milk quality) was evaluated using a mixed model (PROC MIXED) including the fixed effects of treatment and time. The aTTD was assessed with PROC MIXED including the effect of treatment, sampling day, their interaction (treatment × sampling day) and the random effect of animals within the treatment period. Each animal was considered as experimental unit.

For all the parameters a *p*-value ≤ 0.05 was considered statistically significant.

## 3. Results

### 3.1. Trial I: Study of the Effect of the Blend of Essential Oils, Bioflavonoids and Tannins on In Vitro CH_4_ Production

#### 3.1.1. Results of the First Assay

Data regarding the CH_4_ and total gas production after 20 h of incubation, are reported in Table 4.

Overall, the inclusion of the blend of essential oils, bioflavonoids and tannins reduced the production of CH_4_ (*p* = 0.0017) and total gas (*p ≤* 0.001) compared to the Control Test 1. Specifically, CH_4_ production was reduced by the blend, compared to the Control Test 1, with percentages variable from −9.41 in Test 5 (*p* ≤ 0.05) to −20.00 in Test 3 (*p* ≤ 0.05).

Total gas production was reduced compared to the Control Test 1 in all the tests, with percentages variable from −13.84 in the Test 4 to −19.28 in Test 3 (*p* ≤ 0.001).

CH_4_, as percentage of total gas, was similar in each test when compared to the Control Test, but was different between Test 4 and Test 5 (*p* *≤* 0.05).

#### 3.1.2. Results of the Second Assay

Data regarding CH_4_ and total gas production after 16 h and 24 of incubation are reported in Table 5.

Compared to the Control Test 1, monensin sodium (Test 2), after 16 h and 24 h incubation, reduced CH_4_ production by 7.99% and 32.02%, respectively (*p* ≤ 0.001). Total gas production was reduced by monensin sodium by 12.56% and 30.71%, respectively, after 16 h and 24 h of incubation (*p* ≤ 0.001). Furthermore, CH_4_, expressed as the percentage of total gas, was higher after 16 h of incubation (+5.2%; *p* ≤ 0.05), and lower after 24 h (−1.17%; *p* ≤ 0.05) in Test 2 compared to the Control Test 1.

The inclusion of the blend of essential oils, bioflavonoids and tannins reduced CH_4_ production compared to the Control Test 1, with percentages variable from −5.24 in the Test 3 (*p* ≤ 0.05) to −11.92 in Test 5 (*p* ≤ 0.001) at 16 h, and from −10.52% in Test 3 (*p* ≤ 0.001) to −33.26% in Test 5 (*p* ≤ 0.001) at 24 h. Total gas production was reduced by the blend compared to Control Test 1, with percentages variable from −3.15% in Test 3 (*p* ≤ 0.05) to −9.18% in Test 5 (*p ≤* 0.001) after 16 h, and from −8.03% in Test 3 (*p* ≤ 0.001) to −34.86% in Test 5 (*p* ≤ 0.001) after 24 h. Compared to Control Test 1, CH_4_ expressed as the percentage of total gas, was higher in Test 3 (*p* ≤ 0.05) and Test 5 (*p* ≤ 0.05) at 16 h of incubation, and in Test 3 and 4 (*p* ≤ 0.05) at 24 h, but lower in Test 5 (*p* ≤ 0.05).

Compared to monensin sodium, CH_4_ production was higher in Test 3 at 16 h (+5.3%; *p* ≤ 0.05) and 24 h (+35.3; *p* ≤ 0.001) and in Test 4 at 24 h (+14.2%; *p ≤* 0.001), while it was lower in Test 5 after 24 h of incubation (−4.2%; *p* ≤ 0.05).

Compared to monensin sodium, the total gas production was higher in Test 3 at 16 h (+8.4%; *p* ≤ 0.001) and 24 h (+29.1%; *p* ≤ 0.001) and in Test 4 at 24 h (+9.5%; *p ≤* 0.001), while it was lower in Test 5 at 24 h (−5.2%; *p* ≤ 0.05).

Compared to monensin sodium, the percentage of CH_4_ on total gas was lower in Test 3 (−2.9%; *p* ≤ 0.05), Test 4 (−2.6%; *p* ≤ 0.05) and Test 5 (−2.0%; *p* ≤ 0.05) after 16 h of incubation, and higher in Test 3 (+4.7%; *p ≤* 0.001) and Test 4 (+4.2%; *p ≤* 0.001) at 24 h of incubation.

Regarding volatile fatty acids (VFA) (Table 6), acetic and propionic acids were detected in all five tests, while the other acids were absent or present in concentrations lower than the detection limit of 50 mg/L. The inclusion of the blend reduced the production of acetic acid compared to the Control Test 1, with percentages variable from −7.45% in Test 3 (*p* ≤ 0.05), to −72.8% in Test 5 (*p* ≤ 0.001) at 16 h, and from −6.18% in Test 3 to −79.8% in Test 5 (*p* ≤ 0.001) at 24 h. Propionic acid production was increased compared to the Control Test 1 in Test 3 and Test 4 after 16 h (+4.44 and +8.33%; *p* ≤ 0.05 and *p* ≤ 0.001, respectively) and 24 h (+5.15 and +14.43, *p* ≤ 0.05 and *p* ≤ 0.001, respectively), while it was reduced in Test 5 at both 16 h and 24 h (−50.00% and −65.5%, respectively, *p* ≤ 0.001).

Compared to the Control Test 1, monensin sodium also reduced the production of acetic acid at 16 h and 24 h (−16.2% and −27.7%, respectively; *p* ≤ 0.001), while it increased propionic acid at 16 h and 24 h (12.2% and 17.55%, respectively; *p* ≤ 0.001).

Compared to monensin sodium, the blend increased the production of acetate in Test 3 and Test 4 after 16 h (+10.5% and +7.3%, respectively; *p* ≤ 0.001), and 24 h (+31.5% and +4.7%; *p* ≤ 0.001 and *p* < 0.05, respectively), while it was reduced in Test 5 at 16 h and 24 h (−67.5% and −71.7%, respectively; *p* ≤ 0.001). Conversely, the production of propionic acid was lower at 16 h and 24 h in Test 3 (−16.9% and −10.5%, respectively; *p* ≤ 0.001), Test 4 (−3.5% and −2.6%, respectively; *p* ≤ 0.05) and Test 5 (−55.4% and −70.6%, respectively; *p* ≤ 0.001) compared to monensin sodium.

### 3.2. Trial II: In Vivo Study of the Effect of the Blend of Essential Oils, Bioflavonoids, and Tannins on Dairy Cow Performance

#### 3.2.1. Milk Yield, Feed Intake, Feed Conversion Rate, and Milk Quality

**Figure 1:**
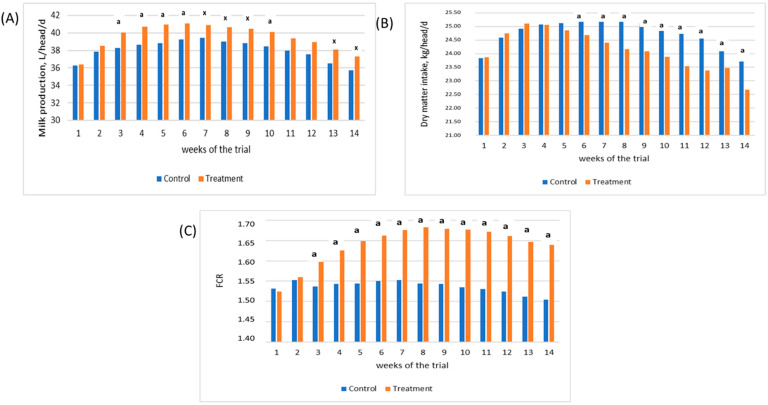
(**A**) Average weekly milk production (kg/head/d) in Control and Treatment groups (a = *p*-value ≤ 0.05; x = *p*-value ≤ 0.1); (**B**) Average weekly dry matter intake (kg/head/d) in Control and Treatment groups (a = *p*-value ≤ 0.05); (**C**) Average weekly feed conversion rate (FCR) in Control and Treatment groups (a = *p*-value ≤ 0.05).

Data for weekly milk yield, feed intake and feed conversion rate for both experimental groups are given in Table 7. The Treatment group showed an increase, during the entire trial, in milk production by 3.8%, equal to 1.40 kg/head/day (*p* ≤ 0.001). Moreover, the ECM was higher in the Treatment group (*p* ≤ 0.001). As shown in Figure 1A, productivity began to differ significantly from the third week of the study in the Treatment group compared to the Control group.

Feed intake and feed conversion rate were also statistically different in the Treatment group (*p* ≤ 0.001). As shown in Figure 1B, dry matter intake was lower in the Treatment group starting from the 6th week of the study. Feed efficiency, as shown in Figure 1C, was similar in the first two weeks of the study but started to be higher in the Treatment group from the third week, resulting in an overall 7.2% improvement in FCR.

Data on milk quality are given in Table 8. Milk fat, proteins, urea and caseins content were not affected by the Treatment, while somatic cell count was lower in the second month (*p* = 0.042) of the study.

Considering health status, all the cows involved in the trial showed good health and no specific issues were highlighted during the whole trial.

#### 3.2.2. Apparent Total Tract Digestibility of the Diet

The average values of chemical characteristics of the diet of the two groups, given in Supplementary Figure S1, showed a good match between the software projection and the chemical analysis of TMR. Supplementary Figure S2 shows the average chemical characteristics of the feces in both Control and Treatment groups. Table 9 shows the average values of the apparent total tract digestibility of the different nutrients in both experimental groups. The Treatment group showed an increase of 23.57% and 1.8% in the aTTD, respectively, of cellulose (*p* ≤ 0.001) and starch (*p* = 0.0023).

## 4. Discussion

Reducing ruminal CH_4_ emissions is critical for dairy cow producers, both to satisfy the societal pressure regarding the environmental impact of dairy products, and to improve overall production efficiency, by shifting ruminal fermentation toward more efficient pathways.

For these purposes, natural compounds such as essential oils, bioflavonoids and tannins have shown promising results [8,18,19,21,30]. However, the results are highly variable, both in in vitro and in vivo studies, depending on the natural compounds used, the dosages and proportions, the duration of the administration and the animals’ characteristics and management. These factors can influence ruminal microflora and its activity, and, consequently, treatment efficacy [8,30].

The in vitro assays performed in the present study were conducted on a standardized anaerobic mud and not on the ruminal fluid that is normally characterized by a highly variable microbiota. In addition, a substrate with a well-known methanogenic potential (anhydrous glucose) was used instead of a highly variable substrate, such as the TMR of dairy cows. Because of these conditions, it is important to emphasize that comparison with other studies of CH_4_ production, evaluated either in vitro on ruminal fluids or in vivo using respiratory chambers, is not strictly feasible.

The results of the present in vitro study showed a significant effect of the investigated blend of essential oils, bioflavonoids and tannins in reducing both total gas and CH_4_ production in comparison with the Control Tests; this was in line with previous in vitro research that has assessed the effect of a similar blend of essential oils but using ruminal fluids [19]. Moreover, in vivo studies performed with similar natural compounds have reported an average CH_4_ reduction of 8%, in agreement with the results obtained in Test 2 and 3 after 20 h and in Test 3 and 4 after 16 and 24 h [8,30].

The improvement in the production of propionic acid highlighted in the present study was in line with the findings of Klop et al. [37] who reported a higher proportion of propionate and lower proportion of acetate in the ruminal fluid of donor cows after in vitro incubation using similar natural compounds. The excessive reduction in volatile fatty acid production observed in the second assay in Test 5 could be attributed to the high concentration of the blend used and to the consequent negative effects on all the bacterial strains, which is consistent with the highest CH_4_ reduction.

Elcoso et al. [30] reported that the reduction in CH_4_ production began to be significant after four weeks of treatment of donor cow ruminal fluid, highlighting that this period may be necessary to modulate the ruminal microbiota activity. It is recognized in the literature that diet supplementation aimed at influencing the microbiota and ruminal fermentation requires a period of at least three weeks to show its effects [21]. In the present study milk production and feed efficiency started to significantly increase, respectively, three and six weeks after treatment as a result of modified and improved activity of the ruminal microbiota after this time frame, confirming that three to six weeks could be considered the required time to modulate the rumina microbiota. Guash et al. [38], in a study concerning the inclusion of essential oils in the diet of dairy cows, reported significant effects starting from the 20th day of administration.

The increase in the milk production of 3.8% highlighted in the present study in the Treatment group is in line with the findings of Belanche et al. [8], who, in a review regarding the effects of similar essential oils, reported an increase of 3.6% in milk yield when the additive was administered for more than four weeks. In contrast, Santos et al. [39], did not report any effect on milk production when similar essential oils were used for four weeks. It seems that the results are mainly dependent on the duration of treatment, in agreement with the previously cited microbiota timing of adaptation [8], and with short-term studies (<4 weeks) showing either no or small (<2%) improvement in milk production, and long-term studies (>4 weeks) showing good (>3%) improvement.

The higher milk production recorded in the present study was combined with lower feed intake and, consequently, with an improved feed conversion rate, as reported also by Elcoso et al. [30] who observed an improvement in the feed conversion index of 2% in a study conducted on dairy cows fed with similar essential oils. Several studies showed an increase in rumen volatile fatty acid production in cattle treated with essential oils, because of more viable and efficient ruminal microbial activity [40,41].

More viable ruminal microbial activity can also lead to increased digestion efficiency [41,42]. The results observed in the present study in terms of aTTD are in line with the findings of Matlup et al. [42] who reported a significant improvement in fiber digestibility in lactating Holstein cows supplemented with coriander essential oil.

This increase in aTTD can be explained by the action exerted by the blend of essential oils, bioflavonoids and tannins on the ruminal microflora. Several studies based on similar natural compounds have shown an increase in the main ruminal populations involved in both structural and nonstructural carbohydrates degradation, such as *Ruminococcaceae* and *Propionic acid* bacteria [20,23,43], with an improvement in propionate production and the acetate:propionate ratio [24].

Moreover, different bibliographical studies, both in vitro and in vivo, have reported inhibitory activity of the essential oils tested in the present study on ruminal methanogenic bacteria, highlighting how this can increase the bioavailability of substrates for other microbial populations that can lead to a greater production of VFA and, consequently, to higher productivity [18,20,21,43].

While milk yield was improved by the treatment, no differences were found in milk quality (protein, fats, caseins, and urea). These results are in line with the findings of Carrazco et al. [19], and Hart et al. [21] who reported no effects of similar natural products on milk parameters. Belance et al. [8], in a comprehensive review, highlighted that the higher milk fat and protein yields reported in some studies were only related to higher milk production, while milk fat and protein percentages were not affected by the treatment. However, Santos et al. [39] reported an increase in the milk fat percentage due to changes in the rumen volatile fatty acid profile induced by essential oil supplementation.

The significant decrease in the milk somatic cell count observed in the present study, two months after treatment, can be related to the anti-inflammatory and antioxidant properties of the natural extracts which are able to stimulate the immune defenses of the mammary gland, reducing the incidence of subclinical mastitis [44]. This hypothesis is supported by the results of Hashemzadeh-Cigari et al. [45] who observed a significant decrease in the somatic cell count and subclinical mastitis in dairy cows supplemented with natural extracts.

## 5. Conclusions

The blend of essential oils, bioflavonoids and tannins used in the present study reduced methane production in the in vitro study from 8 to 22% with optimal concentrations (0.0025–0.005% DM of the pure product in liquid form) at 24 h, and, in the in vivo study, improved milk production, diet digestibility and feed conversion rate.

These results highlight the potential efficacy of natural products, such as essential oils, bioflavonoids and tannins, in improving the production performance of dairy cows and reducing methane production in vitro, that could lead, if further validated in in vivo trials, to a reduction in the environmental footprint of lactating dairy cows.

## Figures and Tables

**Table 1:** Experimental protocol for assay 1 (powder form blend with 10% concentration).

Parameter	Control Test 1	Test 2	Test 3	Test 4	Test 5
Glucose, mg	2000	2000	2000	2000	2000
Blend, mg (% DM_glucose_)	-	0.10 (0.005)	1.00 (0.050)	5.00 (0.250)	10.00 (0.500)

**Table 2:** Experimental protocol for assay 2 (liquid pure form blend).

Parameter	Control Test 1	Test 2	Test 3	Test 4	Test 5
Glucose, mg	2000	2000	2000	2000	2000
Blend, mg (% DM_glucose_)	-	-	0.05 (0.0025)	0.10 (0.005)	0.50 (0.025)
Monensin sodium, mg (% DM_glucose_)	-	0.12 (0.006)	-	-	-

**Table 3:** Nutritional composition and values of the total mixed ration (TMR) used for in vivo study (predicted by Plurimix ^1^, a ration balancing software package).

Feed	kg/head/day, as Fed
Corn silage	18.0
Alfalfa hay	3.0
Raygrass hay	1.6
Wheat silage	8.0
Corn meal	6.4
Soybean meal 44% CP ^2^	4.6
Min vit supplement	0.5
kg/head/d	
As fed, kg	42.10
DM ^3^, kg	23.88
Analysis, % of DM in the TMR	
DM, %	43.30
Energy, Mcal/kg DM	1.61
UFL ^4^/kg DM	0.95
CP	16.27
CF ^5^	2.69
NDF ^6^	33.27
ADF ^7^	22.65
ADL ^8^	4.51
Starch	27.56
Ca ^9^	0.77
P ^10^	0.34

^1^ Plurimix = Fabermatica, Piazza Bruno Pari, 3 GPS: 45.22305 10.25275, 26032 Ostiano (CR); ^2^ CP = crude protein; ^3^ DM = dry matter; ^4^ UFL = feed units for lactation; ^5^ CF = crude fats; ^6^ NDF = neutral detergent fiber; ^7^ ADF = acid detergent fiber; ^8^ ADL = acid detergent lignin; ^9^ Ca = calcium; ^10^ P = phosphorus.

**Table 4:** Production of methane and total gas at 20 h incubation. Different letters in the same column denote significant differences (a,b,c,d *p* ≤ 0.05).

Test	CH_4_ ^1^	Total Gas ^2^	% CH_4_ ^3^
Control Test 1	188.9 ^a^ ± 1.41	304.3 ^a^ ± 1.00	62.1 ^a,b^ ± 1.00
Test 2	162.2 ^b,c^ ± 1.62	250.0 ^d^ ± 1.00	64.8 ^a,b^ ± 1.00
Test 3	151.1 ^c^ ± 1.86	245.6 ^d^ ± 1.00	61.5 ^a,b^ ± 1.00
Test 4	155.6 ^c^ ± 2.10	262.2 ^b^ ± 1.00	59.3 ^b^ ± 1.00
Test 5	171.1 ^b^ ± 2.36	256.5 ^c^ ± 1.00	66.7 ^a^ ± 1.00

Data are presented as least squared means ± standard error of the means (SEM). ^1^ CH_4_ expressed in Nm^3^CH_4_ t_TQ_^−1^ = normal cubic meters of methane per ton of the substrate total mass; ^2^ total gas production expressed in Nm^3^ t_TQ_^−1^ = normal cubic meters of total biogas per ton of the substrate total mass; ^3^ % of total gas production.

**Table 5:** Production of methane and total gas at 16 and 24 h. Different letters in the same column denote significant differences (a,b,c,d,e *p* ≤ 0.05).

Test	CH_4_ ^1^		Total Gas ^2^		% CH_4_ ^3^	
h	16	24	16	24	16	24
Control Test 1	142.7 ^a^ ± 1.00	228.9 ^a^ ± 1.00	236.5 ^a^ ± 1.00	368.9 ^a^ ± 1.00	60.3 ^c^ ± 0.15	62.0 ^b^ ± 0.15
Test 2	131.3 ^b^ ± 1.00	155.6 ^c^ ± 1.00	206.8 ^c^ ± 1.00	255.6 ^d^ ± 1.00	63.5 ^a^ ± 0.15	60.8 ^c^ ± 0.15
Test 3	138.2 ^a^ ± 1.00	210.5 ^b^ ± 1.00	224.^b^ ± 1.00	330.1 ^b^ ± 1.00	61.6 ^b^ ± 0.15	63.7 ^a^ ± 0.15
Test 4	130.3 ^b^ ± 1.00	177.7 ^c^ ± 1.00	210.6 ^c^ ± 1.00	280.2 ^c^ ± 1.00	61.8 ^b^ ± 0.15	63.4 ^a^ ± 0.15
Test 5	129.6 ^b^ ± 1.00	149.1 ^d^ ± 1.00	208.3 ^c^ ± 1.00	246.2 ^e^ ± 1.00	62.2 ^b^ ± 0.15	60.5 ^c^ ±0.15

Data are presented as least squared means ± standard error of the means (SEM). ^1^ CH_4_ expressed in Nm^3^CH_4_ t_TQ_^−1^ = normal cubic meters of methane per ton of the substrate total mass; ^2^ total gas production expressed in Nm^3^ t_TQ_^−1^ = normal cubic meters of total biogas per ton of the substrate total mass; ^3^ % of total gas production.

**Table 6:** Volatile fatty acid concentrations at 16 and 24 h of incubation (in brackets the percentage difference compared to the Control test 1 are reported). Different letters in the same column denote significant differences (a,b,c,d,e *p* ≤ 0.05).

Test	Acetic Acid, mg/L		Propionic Acid, mg/L	
h	16	24	16	24
Control Test 1	228 ^a^ ± 1.00	178 ^a^ ± 1.00	180 ^d^ ± 1.00	194 ^d^ ± 1.00
Test 2	191 ^d^ ± 1.00	127 ^d^ ± 1.00	202 ^a^ ± 1.00	228 ^a^ ± 1.00
Test 3	211 ^b^ ± 1.00	167 ^b^ ± 1.00	188 ^c^ ± 1.00	204 ^c^ ± 1.00
Test 4	205 ^c^ ± 1.00	133 ^c^ ± 1.00	195 ^b^ ± 1.00	222 ^b^ ± 1.00
Test 5	62 ^e^ ± 1.00	36 ^e^ ± 1.00	90 ^e^ ± 1.00	67 ^e^ ± 1.00

Data are presented as least squared means ± standard error of the means (SEM).

**Table 7:** Production performances in the Control and Treatment groups.

	Group		*p* Value
	Control	Treatment	
Production Performance			
Milk yield, kg/head/d	36.90 ± 0.23	38.30 ± 0.23	≤0.001
ECM ^1^, kg/head/d	38.30 ± 0.52	40.2 ± 0.52	≤0.001
DMI ^2^, kg/head/d	24.70 ± 0.08	24.10 ± 0.08	≤0.001
FCR ^3^	1.49 ± 0.01	1.58 ± 0.01	≤0.001

Data are presented as least squared means ± standard error of the means (SEM). ^1^ ECM = energy corrected milk; ^2^ DMI = dry matter intake; ^3^ FCR = feed conversion rate.

**Table 8:** Milk quality parameters in Control and Treatment groups.

	Characteristics of Milk, Month of Study		
	1st Month (August)	2nd Month (September)	3rd Month (October)
		Fat, %	
Control	3.74 ± 0.02	3.70 ± 0.02	3.77 ± 0.02
Treatment	3.79 ± 0.02	3.76 ± 0.02	3.80 ± 0.02
*p*-value	0.185	0.155	0.148
		Proteins, %	
Control	3.31 ± 0.02	3.35 ± 0.02	3.27 ± 0.02
Treatment	3.33 ± 0.02	3.39 ± 0.02	3.25 ± 0.02
*p*-value	0.377	0.690	0.635
		Urea, mg/dL	
Control	21.93 ± 0.23	21.92 ± 0.23	22.28 ± 0.23
Treatment	21.99 ± 0.23	21.83 ± 0.23	22.36 ± 0.23
*p*-value	0.994	0.792	0.690
		Caseins, %	
Control	2.80 ± 0.04	2.99 ± 0.04	2.96 ± 0.04
Treatment	2.79 ± 0.04	2.97 ± 0.04	2.95 ± 0.04
*p*-value	0.821	0.81	0.552
		Somatic cells, x.000	
Control	135.80 ± 3.45	150.09 ± 3.45	144.60 ± 3.45
Treatment	128.22 ± 3.45	141.75 ± 3.45	136.69 ± 3.45
*p*-value	0.078	0.042	0.103

Data are presented as least squared means ± standard error of the means (SEM).

**Table 9:** Apparent total tract digestion of the diet in Control and Treatment groups.

Month	August	September	Octorber	Average	P (g) ^1^	P (m) ^1^	P (g*m) ^1^
Ash, %							
Control	61.63 ± 1.87	61.83 ± 1.87	63.27 ± 1.87	62.24 ± 1.53	0.612	0.654	0.5557
Treatment	61.73 ± 1.87	65.05 ± 1.87	62.41 ± 1.87	63.06 ± 1.53			
*p*-value	0.971	0.271	0.756	0.612			
Crude Fats, %							
Control	69.15 ± 1.63	71.91 ± 1.63	72.44 ± 1.63	71.17 ± 0.94	0.818	0.059	0.672
Treatment	67.85 ± 1.63	72.50 ± 1.63	74.11 ± 1.63	71.49 ± 0.94			
*p*-value	0.593	0.807	0.496	0.818			
Cellulose, %							
Control	43.14 ± 1.23	39.26 ± 1.23	43.24 ± 1.23	41.88 ± 0.71	≤0.001	0.031	0.469
Treatment	51.21 ± 1.23	49.57 ± 1.23	54.46 ± 1.23	51.74 ± 0.71			
*p*-value	0.003	0.001	0.0007	≤0.001			
Hemicellulose, %							
Control	67.66 ± 2.12	67.56 ± 2.12	65.23 ± 2.12	66.81 ± 1.22	0.098	0.672	0.915
Treatment	70.19 ± 2.12	70.88 ± 2.12	69.55 ± 2.12	70.21 ± 1.22			
*p*-value	0.433	0.311	0.20	0.098			
Starch, %							
Control	93.46 ± 0.41	93.09 ± 0.41	93.19 ± 0.41	93.25 ± 0.23	0.0023	0.770	0.397
Treatment	94.47 ± 0.41	95.02 ± 0.41	95.33 ± 0.41	94.94 ± 0.23			
*p*-value	0.13	0.015	0.010	0.0023			
Sugars and Pectins, %							
Control	98.23 ± 0.44	97.92 ± 0.44	98.12 ± 0.44	98.09 ± 0.44	0.0926	0.138	0.314
Treatment	98.25 ± 0.44	96.49 ± 0.44	97.11 ± 0.44	97.28 ± 0.44			
*p*-value	0.981	0.062	0.159	0.092			

Data are presented as least squared means ± standard error of the means (SEM). ^1^ g = effect of the treatment; m = effect of the month; g*m = their interaction.

## Data Availability

The data presented in this study are available on request from the corresponding author.

## Supplementary Materials

The following are available online at https://www.mdpi.com/article/10.3390/ani12060728/s1, Figure S1: Analysis of the composition of the diet, in both the Control and the Treatment groups, done with the portable NIR instrument Polispec, during the trial; Figure S2: Analysis of the composition of the Control and Treatment feces, done with the portable NIR instrument Polispec, during the trial.
