# Supplementary material for: Effect of a Blend of Essential Oils, Bioflavonoids and Tannins on In Vitro Methane Production and In Vivo Production Efficiency in Dairy Cows
Source: Animals (Basel). 2022 Mar 14;12(6):728. doi: 10.3390/ani12060728 (PMC8944839; doi:10.3390/ani12060728)

Supplementary Figure S1 Analysis of the composition of the diet, in both the Control and the Treatment groups, done with the portable NIR instrument Polispec, during the trial

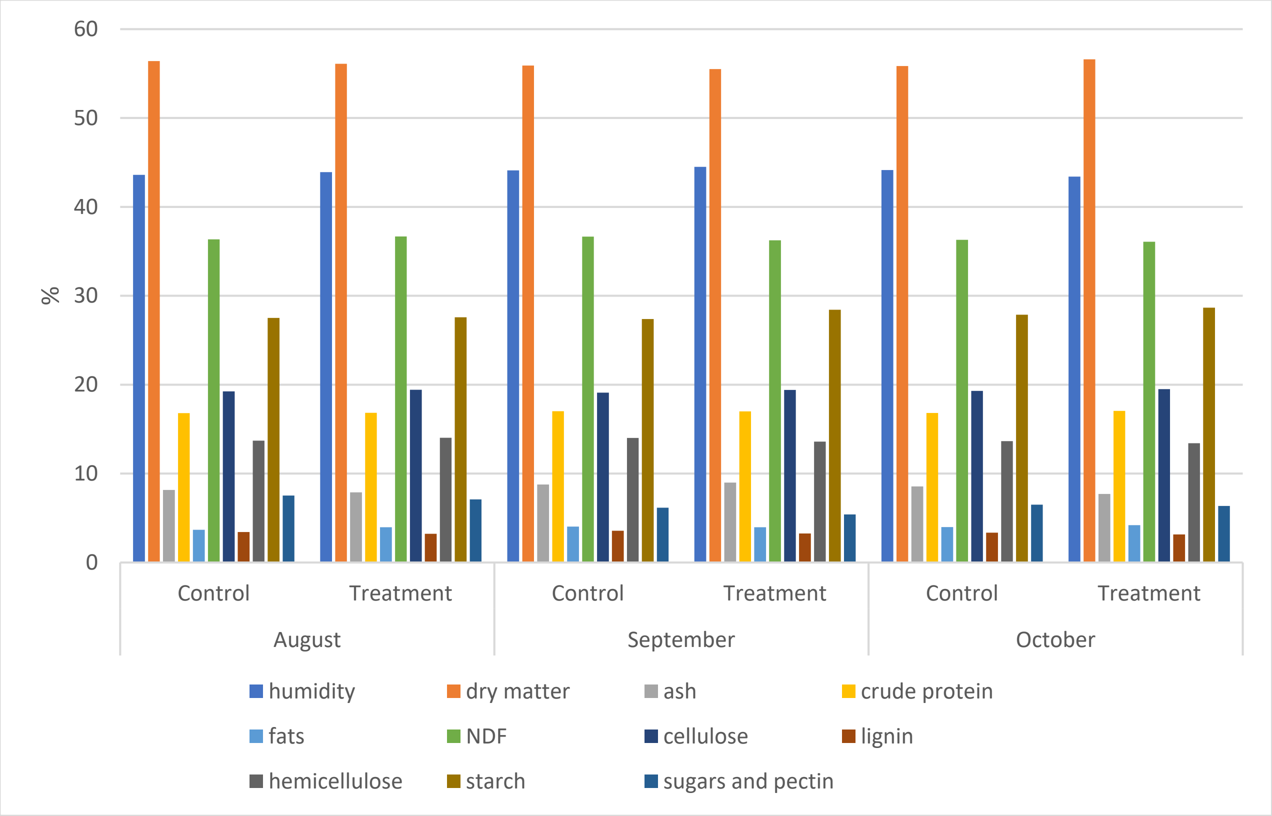

Supplementary Figure S2 Analysis of the composition of the Control and Treatment feces, done with the portable NIR instrument Polispec, during the trial

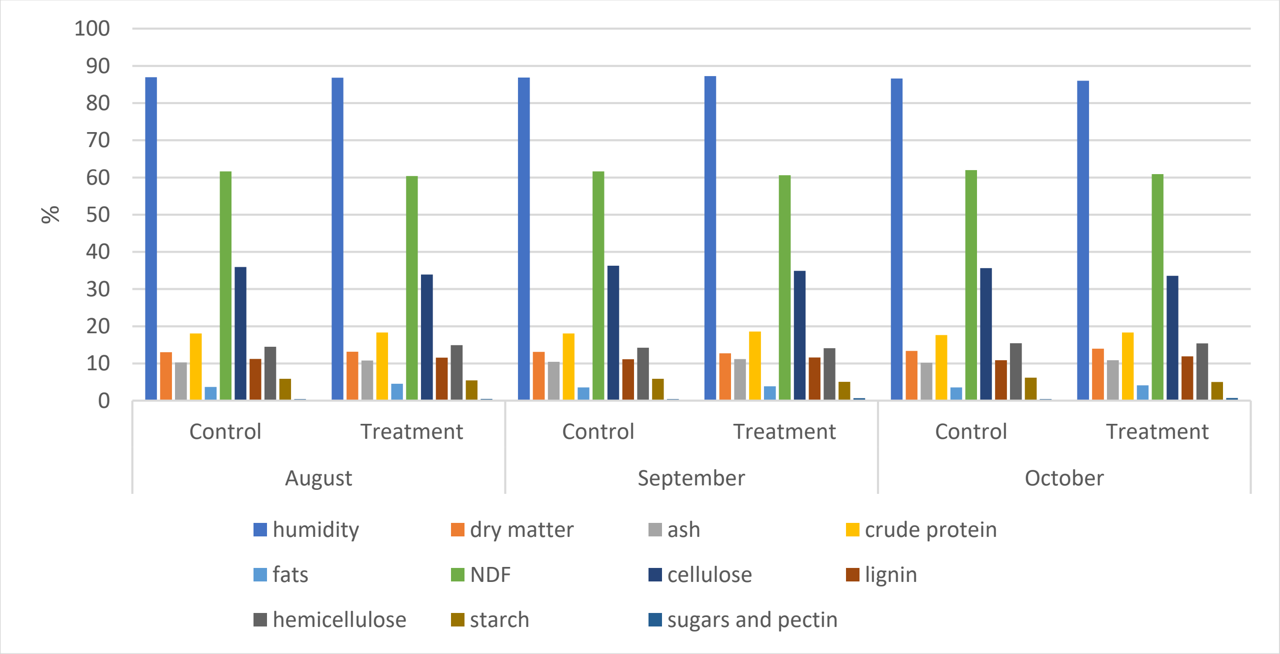

Supplement: Supplementary file 1 [file animals-12-00728-s001.zip › animals-1563225-supplementary.pdf]
